# Compound Fracture of Right Superior and Inferior Maxillæ, with Compound Depressed Fracture of Skull and Hernia Cerebri—Recovery

**Published:** 1880-04

**Authors:** 


*Ashton-Under-Lyne District Infirmary.—Compound Fracture of Right Superior and Inferior Maxillæ, with Compound Depressed Fracture of Skull and Hernia Cerebri; Recovery. (Under the Care of Mr. Hopwood.)*

For the notes of this case we are indebted to Mr. S. J. Rennie, the house-surgeon.

W. McC——, aged twenty-eight, bricksetter, was admitted July 30th, 1879, for injuries. He was engaged in removing the center support of the arch of a brick-kiln, and before he could get out of the way the arch fell, and the patient and another man were buried in the ruins. The arch was about nine feet high from the center to the ground.

On admission the patient had a large ragged wound across the right side of his face, from his nose, one nostril of which was torn open, to his ear, passing beneath the margin of the orbit. There was found to be a compound comminuted fracture of the right superior maxilla, which had evidently been caused by a large stone smashing his face. The line of fracture ran through between the central incisors, and the antrum was opened into. The fracture then ran beneath the orbit to the malar bone, which was comminuted, and the zygomatic arch broken. The coronoid process of the lower jaw was broken off, and there was a depressed fracture of the temporal bone just above the zygoma, from which the brain protruded to about the size of a strawberry.

The coronoid process of the lower jaw, and the broken zygoma were removed, the protruding brain matter was shaved off, and the temporal bone, which was slightly driven in, was easily elevated with the tip of the finger. The wound was then washed out with carbolic lotion, several splinters of bone removed, and the parts brought together by sutures, a drainage-tube being introduced. Carbolic lotion as a dressing was applied, a wedge placed between the teeth to elevate the upper jaw, and the whole secured by a four-tailed bandage. Temperature 99° Fahr; pulse 62, full, strong, regular. Ordered six grains of calomel with six of compound ipecacuanha powder. Head to be shaved, and ice-bag applied.

The patient was perfectly sensible when brought in, and thought he was only slightly hurt. There was no shock, nor had there been any. The pupils were perfectly regular, there was no paralysis of the facial muscles, and the patient expressed himself better as soon as his injuries had been attended to.

Next morning the temperature 98.6°; pulse 62. He felt better. There was some redness about the margin of the wound. Bowels not opened. The calomel and ipecacuanha were repeated; and one-tenth of a grain of tartarized antimony was given every four hours in an effervescing saline mixture; ice to suck; milk and beef-tea. Mouth and nose to be washed out twice daily with Condy's fluid. Evening temperature 99.2°; pulse 64. The bowels were opened once freely. Had one-fourth of a grain of morphia hypodermically.

August 1st.—Morning: Temperature 98.4°; pulse 66. Slept well. Wound looking well. Bowels not opened. Ordered saline aperient mixture. Evening: Temperature 98.2°; pulse 63. Taken milk and beef-tea well. Bowels opened once freely since aperient. Pulse rather irregular and bounding. A quarter of a grain of morphia injected hypodermically.

2nd.—Still feels better. Some redness about neck, but wound looked well. One or two sutures removed. Bowels not opened. Calomel and saline aperient repeated.

3rd.—Morning: Temperature 98.6°; pulse 70, full and compressible. Still improving. Redness about wound less. Wound syringed with Condy's fluid. Bowels opened twice during night. Slept well.

4th.—Better. Wound looking healthy; several sutures removed; dressed as before. Takes milk and beef-tea freely. Tongue clean. Temperature 98.5°; pulse 75. Ordered the calomel and white mixture as before.

6th.—Slept fairly; feels better. Several enlarged glands had made their appearance in his left arm, evidently caused by the irritation of the hypodermic injection; these were painted with iodine.

On the 8th the enlarged glands had disappeared; drainage-tube removed. Wound, from nose half-way, healed, other half granulating, having been kept open by drainage-tube. Tongue clean; bowels regular. Temperature 98.2°; pulse 68. On the 9th he said he felt as well as ever he did in his life. On the 10th the wound was brought together by strapping. Allowed meat, shredded fine, and oatmeal poridge with milk.

14th. Temperature 97.6°; pulse 76. Still progressing favorably ; wound three-quarters healed, rest granulating rapidly.

On the 23rd he was allowed to get out of bed. On the 27th the wound was entirely healed. On Sept. 1st he was convalescent. He had some stiffness on trying to move his lower jaw, and there was some tenderness still during mastication. There is a slight sinking in along the line of fracture, but very little deformity, considering the frightful nature of his injuries.

On Oct. 14th he was seen. He was quite well and working regularly ; there was very little disfigurement.

*Remarks.*—The interesting features of this case are, the enormous amount of injury sustained, with absolutely no symptoms of any moment; and also that it is an illustration of the fact that comparatively large portions of the convolutions of the cerebrum may be removed without any bad results. It shows the value of the careful removal of all, even the minutest, splinters in cases where is a compound comminuted fracture of a thin bone like the wall of the antrum, and the use of frequent syringing of the wound, to remove, as soon as formed, any discharge which might become a source of irritation.

